# Exploring Maternal Resilience among Predominantly Low-Income and Minoritized Women

**DOI:** 10.1007/s10995-025-04044-3

**Published:** 2025-01-27

**Authors:** Fathima Wakeel, Yalitza Corcino-Davis, Samara Everman, Patricia H. Manz, Gabriella Ledis

**Affiliations:** 1 Department of Population Health, College of Health, Lehigh University, Bethlehem, USA; 2 Department of Educational Leadership, College of Education, Lehigh University, Bethlehem, USA; 3 Department of Education & Human Services, College of Education, Lehigh University, Bethlehem, USA; 4 Department of Psychology, College of Arts and Sciences, Lehigh University, Bethlehem, USA

**Keywords:** Maternal resilience, Maternal stress, Social support, Coping, Trauma

## Abstract

**Background:**

Research has increasingly explored maternal resilience or protective factors that enable women to achieve healthier maternal and child outcomes. However, it has not adequately examined maternal resilience using a culturally-relevant, socio-ecological lens or how it may be influenced by early-life stressors and resources. The current study contributes to the literature on maternal resilience by qualitatively exploring the salient multi-level stressors and resources experienced over the lifecourse by predominantly low-income and minoritized women.

**Procedures:**

Data are from 19 women who were either adult mothers with children under 18 years of age living at home or reproductive-aged. Respondents completed semi-structured interviews, and grounded theory analyses identified themes related to stressors experienced and resilience resources utilized over the lifespan.

**Results:**

Four domains relating to maternal stress and resilience, including *stressors* (caregiving stress, family conflict, and deaths in the family), *traumas* (abandonment, domestic violence, child abuse, and substance misuse), *coping mechanisms* (positive mindset, faith, activities, and movement, healthy eating, self-regulation, and self-love/care), and *supports* (family, friends, spouses/partners, community members, and religious institutions), emerged from the data. Familial relationships were perceived as the most significant support as well as the biggest source of stress and trauma.

**Conclusions:**

As proposed by the socio-ecological framework, our findings suggest that components of maternal resilience exist at the individual (i.e., positive mindset, faith, self-regulation, self-love, and positive health behaviors), interpersonal (i.e., support from family, friends, and partner), and community (i.e., support from community members and religious organizations) levels.

## Significance

Though research has increasingly explored maternal resilience, the literature has not adequately examined it using a culturally-relevant, socio-ecological lens or how it may be influenced by early-life stressors and resources. This study contributes to the literature by exploring the salient stressors and resources experienced over the lifecourse by predominantly low-income and minoritized women. The findings suggest that, as proposed by the socio-ecological framework, maternal resilience components exist at the individual (positive mindset, faith, self-regulation, self-love, and positive health behaviors), interpersonal (support from family, friends, and partner), and community (support from community members and religious organizations) levels.

## Introduction

Extant literature indicates that differential maternal exposures to stressful life events and chronic stress (i.e., interpersonal and systemic racism) before and during pregnancy contribute to maternal and child health (MCH) disparities, disproportionately impacting low-income, minoritized, and other historically marginalized groups (Lu & Halfon, 2003). Though previous work largely focused on maternal stress and other contributors to negative MCH outcomes, research has increasingly explored maternal resilience or protective factors that enable women to better adapt or reduce their exposure to stress. Though maternal resilience has been conceptualized and measured in many ways, the literature has not adequately examined it using a culturally-relevant, socio-ecological lens (i.e., considering factors and processes beyond individual-level traits) or how it may be influenced by early-life factors.

Existing scales (Connor & Davidson, 2003; Wagnild & Young, 1993) have primarily conceptualized maternal resilience as individual traits, such as personal competence and trusting one’s instincts, that enable one’s adaptation to adversity; some of these traits are stable and likely not amenable to interventions. Further, though some maternal resilience measures (Connor & Davidson, 2003; Wakeel et al., 2013) have incorporated interpersonal and community-level resources, such as social network and neighborhood support, they have not fully integrated support systems and other protective factors at the interpersonal (e.g., partner and family), community (e.g., neighbors and faith-based institutions), and systemic (e.g., high-quality education, food, and healthcare) levels into the conceptualization of maternal resilience. The paradigm of individual traits driving resilience unjustly demands individuals, who may lack the opportunities to develop these traits and be more vulnerable to suboptimal or even violent systems, to be resilient, rather than leveraging and developing supports at the interpersonal, community, and macrosystem levels.

Most research on maternal resilience has focused solely on the perinatal period. Less research has examined how early life stressors and resources may shape maternal resilience and how resilience during pregnancy and motherhood can impact long-term outcomes. According to Halfon & Hochstein’s (2002) Life Course Health Development framework, health is determined by cumulative exposures to risky and protective behavioral, social, economic, environmental, and cultural factors over the lifecourse. As maternal resilience is likely the product of interactions among multi-level protective factors experienced over the lifecourse, it cannot be examined in isolation during the perinatal period. Therefore, it is also prudent to broaden the conceptualization of maternal resilience to encompass the experiences of reproductive-aged women, not mothers alone.

Minimal research has focused on exploring maternal resilience among women who are at higher risk for stress and poor health outcomes, including minoritized women. In their noteworthy critical literature review of resilience and adverse obstetric outcomes among Black, Hispanic, and Latina women, Sumbul et al. (2020) conceptualize resilience as a process comprising dynamic interrelationships between multiple ecological levels, including the individual embodiment of stress and discrimination; capacity development through personal and social supports; entitlement of one’s worth and belonging; resistance as a mechanism to improve outcomes by ensuring equity in historically marginalized communities; and the broader contextualization of resilience in historical and structural inequities.

The current study draws on Sumbul et al.’s work and contributes to the maternal resilience literature by qualitatively exploring the multi-level stressors and resources experienced over the lifecourse by predominantly low-income and minoritized reproductive-aged women. This study’s findings elucidate the culturally-relevant components of maternal resilience and inform the development of interventions to help women, particularly those who experience greater stress, garner resilience resources across the lifecourse and ultimately achieve healthier long-term outcomes.

## Methods

Researchers followed the Consolidated Criteria for Reporting Qualitative Studies (COREQ) checklist to ensure all relevant methods and findings were included in the following section (Tong et al., 2007). The study was conducted by five female researchers: two PhDs, one doctoral student, and two undergraduates with expertise in health and education. The first three authors are women of color.

### Sample

Participants included 19 women (M = 41 years), who reside in a region comprising sizable populations of low-income and minoritized (predominantly Latine) individuals (Table 1). Seventeen (89%) were mothers. Many women (*n* = 15; 79%) identified as a racial/ethnic minority, with 58% identifying as Latine (*n* = 11) and 47% (*n* = 9) reporting Spanish as their native language. Most lived in households with incomes below $20,000 (*n* = 12; 63%).

**Table 1:** Description of sample by Sociodemographic Characteristics (*n* = 19)

Sociodemographic Characteristics	Categories	Participant Responses	
		*N*	%
Age			
	18–24	1	5
	25–34	4	21
	35–39	3	16
	40–44	5	26
	45+	6	32
Race/ethnicity			
	White	4	21
	Black	2	11
	Hispanic	11	58
	Other	2	11
Marital status			
	Single	6	32
	Married	8	42
	Divorced/Widowed	3	16
	Cohabitating	2	11
Have children		17	90
Education level			
	Less than high school	4	21
	High school/some college	7	37
	Associate’s/technical degree	3	16
	Bachelor’s degree or higher	5	26
Income levels^a^			
	Less than $20,000	12	63
	$20,000-$39,000	2	11
	More than $40,000	4	21
Primary language			
	English	10	53
	Spanish	9	47

^*a*^ *One participant declined to answer*

### Procedures

Data were collected through 30-45-minute semi-structured interviews. Researchers recruited a convenience sample through a social service agency, the local school district, and word of mouth. Women were included in the study if they were either reproductive-aged (18–44) or adult mothers with children under 18 living at home. A bilingual researcher conducted the interviews in person or via video conferencing in the participant’s language of preference (English/Spanish). Respondents provided informed consent before participating and received a $20 gift card incentive. This study was approved by the [Blinded for review] Institutional Review Board.

### Measures

The interview guide questions (Appendix A) were formulated based on a literature review on maternal resilience, stress, and health (Connor & Davidson, 2003; Montoya-Williams et al., 2020; Wakeel et al., 2013). Women were queried about childhood and adulthood stressors and the personal strengths and multi-level support systems that helped them through those stressors.

### Analytical Approach

Transcripts were coded implementing a hybrid deductive and inductive thematic analysis approach (Fereday & Muir-Cochrane, 2006). A preliminary codebook was created using the interview guide based on the socio-ecological framework employed in the resilience literature. Two researchers tested the initial codebook by coding three interviews to ensure reliability and negotiated code consensus when needed. The remaining interviews were coded using the revised codebook. Inductive codes were also assigned to excerpts that described new codes not included in the original codebook. Researchers linked codes to eliminate redundancy, identified categories and subcategories, and reviewed all excerpts to ensure the data reflected the categories and subcategories. Then, the auditor and the two researchers iteratively reviewed and validated the categories, moving major categories under umbrella domains and collapsing smaller subcategories into larger categories.

## Results

We identified four domains relating to maternal stress and resilience, including *stressors*, *traumas*, *personal strengths and coping mechanisms*, and *interpersonal and community-level supports* (Tables 2 and 3).

**Table 2:** Themes and quotes related to maternal stressors and traumas (*n* = 19)

Domain	Subcategory	Participants Responses		Participant Quotes
		N	%	
Stressors	Caring for loved ones	14	74	“My oldest daughter…has an autoimmune disease, so that’s stressful, just navigating all of that stuff with the doctors.” “She’s got lead poisoning. She eats the wall, the wood, the paint, and she’s anemic…She hardly eats and so it makes it harder for me because she doesn’t eat rice or anything.”
	Family conflicts	13	68	“I stopped trying to affiliate with my family for a while because all they did was crucify me, tell me I wasn’t going to be this, point fingers at me.” “She supports me, but at the same time, she does not because she is always fighting with me.”
	Death	11	58	“I’m still grieving from my first kid’s father. That was very traumatic for me, and just losing my mom. And I had to take care of when she was sick. I take care of my dad when he was sick. I was just like, little old me.” “My husband suffered from…sickle cell. He had seizures a lot, so taking care of him.did become stressful. Then to lose him, it became even more stressful.” “My mom was battling cancer. She got COVID, and that’s what killed her. That was a big pain.”
	Migration	9	47	“Last year, in December, when my husband and son decided to come, it was just too much, too much stress.” “I left my oldest son with my mom because, when one comes here, you can’t come with…one has to settle first.”
	Children	8	42	“And sometimes they are stressful, too, because you think, a lot of people think when kids become grown, it’s more easier, but it’s not.” “Trying to take care of two kids and my daughter, she has to get tested because she might have autism. So, it’s pretty hard trying to cope with that.”
	Financial burdens	7	37	“Right now, I’m trying to figure out how to pay the bills.” “They say that money doesn’t bring happiness. For me, it does because money gives you health insurance, schools, studies, a house, a car. If there is a lack of money, if there is a shortage of money, it will be a conflict.”
	Housing insecurity	7	37	“Today, I went to donate blood to get the…$100 to pay the rent.” “It’s been stressful because I went to another shelter for pregnant moms.and I got kicked out.”
	Employment insecurity	7	37	“I have lost my job and things are getting worse.” I told them, ‘I’m going to pay you back when I start working,’ even though I don’t have a stable job or anything.”
Traumas	Abandonment	9	47	“When I seen the back of my father’s head leave me. I will tell anybody this till the day that I die. What traumatized me the most as a child.” “Five or six year. From then on, I didn’t know what it was like to live with my mother. I knew what it was like to live with my grandmother,…with an aunt, with a niece,.then older siblings.Not everybody pays attention to the care of a child.” “He moved out one day when I was at work. He didn’t tell me. He didn’t tell the kids. He just moved, and I kind of cracked.”
	Domestic violence	7	37	“Very stressful. I even lost a pair of twins because of it.” “I was abused pretty bad. I had to get out of that relationship. My daughter, I had to give her to his mom because I had to get help. I couldn’t cope.”
	Child abuse	6	32	“A stressful, horrible thing, I lived with that for about five, ten years with that, that I couldn’t have sex with anyone afterward, because I felt like I…it covered me up. It was a traumatic, stressful thing.” “He tried to apologize for what he did to me. And I showed my mom the messages, and my mom was like, ‘I’m sorry’…Yet you still don’t believe me…you don’t want to comfort me.”
	Substance use	6	32	“I have an alcohol problem. I’m not going to deny it…it’s them, the situation I have at home.” “I was drinking every day. I was doing coke again like it was nothing. I was like ‘Wow, I’m doing this like nothing’ because the stress was too much.

**Table 3:** Themes and quotes related to maternal coping mechanisms and supports

Domain	Subcategory	Participants Responses		Participant Quotes
		N	%	
Personal strengths and coping mechanisms	Positive mindset	15	79	“To focus on, to focus on that everything is going to be okay. And even if I don’t have a plate of food, I say, ‘I’m going to have it.’ To always be positive.” “I tell myself that I am strong. I can do it.”
	Faith	13	68	“Pray. Got me a long way… It does not seem like much. But when you tap into it and accept it, it helps a lot.” “God has definitely opened a lot of doors.” “God is the only one who keeps me strong.”
	Interacting with others	13	68	“And when you feel like that, talk. You sit outside and look for conversation, because that frees you…Because if you keep everything inside, it makes you sick.” “Talking to someone that actually calms you down.”
	Movement	11	58	“Dancing, taking walks, or talking to myself at least 15, 20 minutes to a half an hour.” “It actually feels good to walk outside.”
	Activities	11	58	“I like to listen to Audibles, reading, self-pampering, hot bath, whatever the case may be, and just maybe some good music that’ll put me in a good mood.”
	Healthy eating	10	53	“Eating well, healthy, what I eat. I eat fruits, I eat salads, I make my own meats.” “You have to take care of yourself because they (food) make you dizzy and nauseous…The nutritionist told me it was because of the fat, the tortilla, the soda, the coffee, and I had to give it all up.”
	Self-regulation	9	47	“I always come back and forgive him…and (he) does the same thing. But now, this time, I don’t think so.” “Everybody, what I realized, is not deserving of that because they don’t have those same intentions, and that became a stress for me…I had to let that go.”
	Self-love / self-care	8	42	“I feel like a strength that I bring now that I didn’t before was that I’ve learned to have a voice for myself.so I can protect myself.” “I think now my strength that I’m working on in times of stress is being better at articulating what I need.”
Interpersonal and Community-level Supports	Family	19	100	“I can tell you that my family was number one, the strongest, deepest support was family.” “I needed to move, and I didn’t have anymore because I wasn’t working. I told them if they could help me because of my situation, and they supported me. They (family) said, ‘Well, you spend at least $3,000,’…and they each offered me a part.”
	Friendships	16	84	“They listen to me. They give me advice.” “I feel a lot safer just being me and not having to put on this persona or fit into whatever category…I do feel more safe in that way, and I also feel more comfortable just letting it all out and not having to worry about the judgment that comes with certain opinions, or thoughts, or feelings.”
	Partner	10	53	“Right now, the supportive people in my life have been my partner, who has been there for me, not give up no matter what.” “He’s probably like my main person.” “My husband is one of those people that whatever I do, everything is fine. He supports me emotionally.”
	Community members	9	47	“It has been a great blessing because I have lived there for 12 years, and…they are very close, and they are always greeting you, how are you, if they have things, they call you.” “It’s kind of scary to be a woman and to be home alone. Even though I have my 16-year-old son, it’s still. But I feel comforted that there’re five women around me…we watch out for each other.”
	Religious institutions	6	31	“About my pastor? Well, I have spiritual, emotional, and comforting support.” The community where I am is very supportive because we do a lot of activities…there’s a lot of support there and one feels very well in God’s hands.”

### Stressors

The most salient stressors entailed family relations, including caring for loved ones, conflicts, and death (Table 2). Almost three-quarters of participants reported caring for loved ones, including children, parents, partners, and extended family, as the biggest stressor. Some participants described the stress of caring for their children with physical, cognitive, or mental health challenges. One participant explained the stress associated with caring for her child who “got lead poisoning” and is “anemic” because “she doesn’t eat rice or anything.” Others discussed stressors associated with caring for parents, such as a participant who described the stress of caring for her mother with Parkinson’s disease until she passed away.

Family conflict was the second-highest reported stressor. Participants frequently discussed being simultaneously stressed and supported by family. One participant stated, “She (mother) supports me, but at the same time, she does not because she is always fighting with me.”

Over half of participants reported that the death of a loved one was a stressful event. Coinciding with the experience of loss, participants acknowledged stress occurring before death while caring for loved ones as well as grieving following their loss. One participant’s “husband suffered from…sickle cell. He had seizures a lot, so taking care of him…did become stressful. Then to lose him, it became even more stressful.” Some participants dealt with multiple deaths of loved ones that severely influenced their stress levels.

### Trauma

Participants reported exposure to traumatic events, including abandonment, domestic violence, child abuse, and substance use. Almost half of the participants reported that abandonment significantly contributed to childhood and adult stress. One participant attributed her current feelings of being “off-balance” and not knowing “the direction I want to go in,” to being abandoned by her father in childhood. Another discussed the traumatic experiences of being abandoned by her partner. She explained, “he moved out one day when I was at work. He didn’t tell me. He didn’t tell the kids. He just moved. And I kind of cracked.”

Participants also shared incidences of enacting, witnessing, or experiencing domestic violence. Most women witnessed domestic violence as a child or were adult victims, placing protection orders against their partners to ensure the safety of their children and themselves. One participant shared, “I even lost a pair of twins because of it”.

About a third of the women shared experiences of childhood physical or sexual abuse. Women spoke about being sexually abused as children and the stress of keeping sexual abuse a secret or experiences of being invalidated when they told an adult. One woman revealed, “He tried to apologize for what he did to me. And I showed my mom the messages, and my mom was like, ‘I’m sorry.‘…Yet, you still don’t believe me…you don’t want to comfort me.” Childhood sexual abuse also negatively affected their romantic relationships. One participant noted, “I couldn’t have sex with anyone afterward because I felt like it covered me up. It was a traumatic, stressful thing.” Additionally, some participants turned to substances to cope or felt stress because of family members’ substance use. One participant admitted, “I have an alcohol problem. I’m not going to deny it… it’s them, the situation I have at home.”

### Personal Strengths and Coping Mechanisms

Women identified multiple personal strengths and coping mechanisms to address stressors. Most participants reported having a positive mindset as an important personal strength. A positive mindset included positive thinking (i.e., telling “myself that I am strong. I can do it”), feelings of motivation and agency, and pride in themselves or others.

Participants also reported faith, interacting with others, movement, activities, being in nature, and healthy eating as positive coping mechanisms. Most women identified their faith as a source of comfort and support. Participants shared that “a higher power is going to help me through this,” “God has definitely opened a lot of doors,” or “God is the only one who keeps me strong.” Notably, not all who identified faith as support associated faith with involvement in a religious institution.

Additionally, women identified interacting with others as a coping strategy. One explained that “talking to someone that actually calms you down.” Other coping mechanisms included movement, activities, and healthy eating. Movement, including exercise, helped women release physical and mental stress. Activities such as journaling and listening to music helped participants relax and process their situations.

Moreover, participants reported self-regulation and self-care as tools utilized to decrease stress and help cope with challenging situations. Some women practiced self-regulation by avoiding unhealthy habits, such as using substances and establishing boundaries with family and friends. A participant discussed establishing boundaries with her abusive partner, “I always come back and forgive him…and (he) does the same thing. But now, this time, I don’t think so.” Women also practiced self-love/care by finding their voice. One participant stated, “I also feel like a strength that I bring now that I didn’t before was that I’ve learned to have a voice for myself. So I can protect myself.” Others demonstrated self-love/care by prioritizing themselves; one woman stated, “I have a habit of putting others first versus myself. And I have to be okay with saying, well, no, let me put me first and be okay with it.”

### Interpersonal and community-level Supports

Family, friends, partners, community members, and faith-based institutions supported participants. All participants identified family as a source of support during stressful times; parents were a significant source of emotional support. One participant shared that her mom and mother-in-law provided emotional support and kept her “strong” while she cared for her hospitalized husband. Family also helped in practical ways, such as financially and with child-rearing. One participant’s extended family “each offered me a part” of $3,000 to help sustain her and her children while she was unemployed. Another participant’s sisters helped raise her when her parents abandoned her as a child.

Friendships were the second most significant source of support. Friends included childhood friends, coworkers, and platonic male and female friends. Women reported that their friends “listen to me. They give me advice.” and “I feel a lot safer just being me and not having to put on this persona or fit into whatever category.” Friends also provided practical help, such as food, shelter, and childcare. For example, a friend allowed a participant and her children to live in her home when the participant was unhoused.

Over half of the participants identified partners as significant sources of support and shared that their partners supplied emotional and practical support. Women shared that their partners “supports me emotionally” and encourage them to “keep my head up,” and “not to give up, no matter what.” Partners also supported participants with advice and child-rearing and household tasks. Not all participants had partners, so the significance of partner support may be underestimated.

Almost half of the participants reported receiving community support, ranging from friendly greetings and conversations to help during difficult situations. A participant explained that she and her neighbors support each other’s safety; “It’s kind of scary to be a woman and to be home alone. Even though I have my 16-year-old son, it’s still. But I feel comforted that there are five women around me…we watch out for each other.” Additionally, some also acknowledged that their religious community provides “spiritual, emotional, and comforting support.”

## Discussion

This study’s findings contribute to the maternal resilience literature by elucidating the salient stressors and resources that predominantly low-income and minoritized women may experience over the lifecourse. Participants perceived familial relationships as their most significant support; however, family-related stressors, including caregiving stress, family conflict, and deaths in the family, were perceived as the most stressful and were often the source of traumas, including being abandoned during childhood or adulthood or experiencing domestic violence, child abuse, or substance use. Prevalent personal strengths and coping mechanisms included a positive mindset, faith, activities and movement, healthy eating, self-regulation, and self-love/care. Along with family, leading sources of interpersonal and community-level support included friends, partners, community members, and faith-based institutions.

Our finding demonstrating family as the most salient support for women is widely supported in national and international studies (Shishehgar et al., 2015; Zhou & Taylor, 2022). Research (Campos et al., 2014; Stanhope et al., 2021) indicates that this trend may be more prevalent among Latinas when compared to other racial and ethnic groups. However, as suggested in our findings and extant research (Sebri et al., 2021), *support sources may not always be supportive*, and low-quality support may be associated with increased stress and adverse health outcomes. Negative social support may introduce demands and conflict (Sebri et al., 2021) or result in concerns about obligatory reciprocity, especially among low-socioeconomic status families (Offer, 2012). Families may also entail “emotional interference or control” (Zhou & Taylor, 2022; pg. 6). Moreover, though the family may not be a source of negative social support, it may involve caregiving stress or family illness or death, as shown in our study and the literature (Stanhope et al., 2021).

Further, many participants experienced significant traumas across the lifecourse, ranging from physical and sexual abuse to abandonment. Trauma can negatively influence women’s reproductive health, contributing to miscarriages and fertility issues (Wamser-Nanney, 2022). Based on her weathering hypothesis, Geronimus and colleagues (2015) found that individuals who are exposed to traumas and chronic stressors, such as racism, discrimination, and other adverse social and environmental exposures, over the lifecourse may experience accelerated aging, disproportionately impacting minoritized women. However, we posit that strong support systems and other resilience resources may mitigate some of these effects.

Our findings suggest that components of maternal resilience exist at the individual, interpersonal, and community levels, as proposed by the socio-ecological framework. At the individual level, respondents reported internal resources, such as a positive mindset, faith, self-regulation, and self-love, and positive health behaviors, including stress-relieving activities and healthy eating, as ways to adapt and even thrive during stressful experiences. The literature has incorporated related constructs such as positive acceptance of change (Connor & Davidson, 2003), self-acceptance (Wagnild & Young, 1993), and sense of control (Beardslee, 1989) into the conceptualization and measurement of resilience. Further, spirituality has been shown to improve mental health outcomes and quality of life among pregnant women (Piccinini et al., 2021). Additionally, though engaging in healthy stress-relieving activities and healthy eating have been linked to positive outcomes among pregnant women and women of color (Page, 2004), it is unclear whether these behaviors are components of maternal resilience or positive coping mechanisms that individuals who possess characteristics such as a positive mindset may be more likely to adopt.

The literature widely corroborates our findings that social networks are instrumental sources of interpersonal resources for women (Rini et al., 2006). However, though partner support has been associated with reduced prenatal stress and anxiety (Martin & Brock, 2023; Rini et al., 2006), positive maternal health behaviors in the perinatal period (Cohen et al., 2016), and increased mother-child attachment (Martin & Brock, 2023), there is limited research on the link between partner support and maternal stress among minoritized and immigrant women, including how this relationship may differ by level of acculturation (Guendelman et al., 2001).

While the literature primarily focuses on the positive impacts of proximal supports and individual attributes on maternal health, our findings suggest that some women may benefit from community-level resources. Some studies have demonstrated the association between neighborhood social capital and reduced maternal stress (Zhang et al., 2015). Further, there is no known research explicitly focusing on the relationship between faith-based institutional support and maternal stress. However, there is substantial but mixed evidence regarding the link between religiosity and maternal stress, with some research demonstrating protective effects on parenting stress among single mothers (Petts, 2012) and postpartum depression among African-American mothers (Keefe et al., 2016), but other research indicating increased perceived stress among English-speaking, but not Spanish-speaking, pregnant and postpartum mothers (Mann et al., 2010).

### Limitations

There are some potential limitations to consider when interpreting our findings. First, the participants may not be representative of women residing in the region due to convenience sampling. Though our sample was largely low-income and minoritized, our findings may not fully capture the diverse range of experiences of these groups. Second, it is unknown if virtual participants had a similar level of privacy as in-person participants; inadequate privacy could have impacted their ability to answer questions truthfully. However, all interviews were scheduled in advance, allowing participants to secure a private location. Third, women’s responses regarding previous experiences were potentially biased by current experiences. For example, a salient childhood stressor or resource may not be perceived as significant or relevant in women’s current contexts.

### Implications and Future Directions

Despite these potential limitations, this study has significant implications for research and interventions. It builds upon the limited existing research (Connor & Davidson, 2003; Montoya-Williams et al., 2020; Wakeel et al., 2013) and provides further support for the conceptualization and measurement of maternal resilience as a multidimensional construct comprising individual, interpersonal, and community-level resources. As this conceptualization of maternal resilience shifts beyond the extant unjust focus on stable individual traits to encompass modifiable and culturally-relevant individual-level and contextual factors, these findings inform the development of public health and clinical interventions that cultivate these resources for women, especially those at highest risk for adverse health outcomes. During prenatal, postpartum, and well-child visits, clinical providers could comprehensively screen women for the types and quality of social support they receive, encourage women to involve family, friends, and community-based allies in their care, and collaboratively develop a strong support system comprising emotional, instrumental, and informational supports (Hawkins et al., 2021).

Our findings further suggest the importance of developing community-based support groups for pregnant and postpartum women. This approach would move beyond widely-used strategies such as group prenatal care to provide culturally-tailored programs that incorporate community-based supports, including mental health resources, in non-clinical settings. Research (Potharst et al., 2022) suggests that mindfulness interventions in non-clinical settings may effectively reduce maternal stress. Additionally, as previous stressors and traumas significantly impact women’s current health, multiple stakeholders, including clinicians, school-based providers, and community-based supports, must be trained to identify signs of trauma and mental health issues and intervene during critical and sensitive periods, including childhood, adolescence, and the four trimesters.

Our findings also have important research implications. As this study provides a preliminary understanding of the salient stressors and multi-level maternal resilience resources that women experience across the lifecourse, validation research should examine the relevance and relative importance of these resources among additional women in the same population and women in other culturally and socioeconomically diverse populations. Studies should also explore how potential broader systemic resilience resources, including cultural resilience and positive determinants of health, may reduce women’s exposure to stressors stemming from inequitable systems and help them better adapt or even thrive during adverse circumstances. Further, longitudinal research is needed to determine which resilience resources may address early-life stressors, thereby informing the development of early interventions that help optimize long-term MCH outcomes.

## Data Availability

Data is available from the authors upon request.

## Appendix A

### Stress Coping, and Reproductive Health Interview Guide

PREAMBLE: I am interested in learning about the types of things that have helped women get through stressful times and achieve good health. Good health can include many things, such as good overall health, having healthy pregnancies and babies, good physical health, good mental health, etc.

1. First, I would like to know about the people in your life. This could be your partner, family member(s), friend(s), support group, co-worker(s), etc. Who gives you support on a regular basis?

Probes: In which kind of situations are these people helpful? If she mentions her family, ask if her family lives near her. At the end, also probe to ask if there is anyone else they would like to mention.

2) Now, I would like to know more about your community. Community can mean different things to you, such as your neighborhood, your extended family, members of your religious institution, etc. How does your community support you?
3) Have you experienced any events in your adult life that have been particularly stressful? If so, could you please tell me more about that, if you feel comfortable doing so? Probe: Examples of stressful life experiences may include divorce or separation from a partner, migrating to a new country, death of a loved one, intimate partner violence, food insecurity, homelessness, etc.
4) What helped you through this stress?

Probes: What kinds of things did you count on to help you to deal with this stress? At the end, also probe to ask if there is anyone/anything else they would like to mention.

5) Did you experience any events in your childhood that were particularly stressful? If so, could you please tell me more about that, if you feel comfortable doing so? Probe: Examples of stressful childhood experiences may include one’s parents getting divorced, death of a loved one, abuse, neglect, food insecurity, homelessness, etc.
6) What had helped you through the stress you experienced during childhood?
7) What are the personal strengths that you bring to stressful or difficult situations (if not mentioned earlier)?

Probes: What personal resources do you use to cope with stressful or difficult situations?

8) Now that we have talked about the different types of things that have helped you in your life, what do you think is the most important thing to help you achieve good health?

### After the Qualitative Portion, ask the Following Quantitative Questions

1. What is your age?
2. Which one of these groups best describes your racial background?

White, Black, Asian/Pacific Islander, Spanish or Hispanic, or other.

3) What is the highest year of school you completed?

Less than high school, high school diploma or GED, some college/associate degree, bachelor’s degree, master’s degree, or more.

4) If you don’t mind telling me, what is your approximate annual household income?

Less than $20,000, $20,000-$39,000, $40,000-$59,000, $60,000-$79,000, $80,000-$99,000, more than $100,000.

5) Are you employed? If so, what do you do?
6) What is your current marital status?

Married, separated/divorced, widowed, single/never married, or cohabiting.

7) Where were you born?
8) If not born in the US, how long have you lived in the US?
9) How many times have you given birth before?
10) If you have been pregnant before, have you experienced any of the following outcomes?

Miscarriage, pregnancy complications (e.g., preeclampsia/gestational hypertension, gestational diabetes, premature rupture of membranes, bacterial vaginosis, fetal growth restriction, bladder or kidney infection), stillbirth, preterm delivery, low birth weight baby, small-for-gestational-age.

11) Do you have any current medical conditions? Examples: high blood pressure, diabetes, anemia, etc.
